# Congenital heart disease prolongs the time to full enteral feeding in neonates with abdominal operation: a dual-center, propensity score-matched retrospective cohort study

**DOI:** 10.1186/s13052-026-02291-w

**Published:** 2026-06-11

**Authors:** Yao Liu, Jing Guo, Yiwen Tan, Yingyuan Wang, Xiying Xiang, Juan Du, Wenqing Kang, Mingyan Hei

**Affiliations:** 1 Beijing Children’s Hospital, Capital Medical University, Beijing, China; 2 Zhengzhou Children’s Hospital Affiliated to Zhengzhou University, Zhengzhou, China; 3 Key Laboratory of Major Pediatric Diseases Research (Ministry of Education), Beijing, China; 4 National Center of Children’s Health, Beijing, 100045 China

**Keywords:** Infant, Newborn, Congenital heart disease, Abdomen, Operation, Feeding

## Abstract

**Background:**

Congenital heart disease (CHD) may cause intestinal hypoperfusion and feeding intolerance. The aim of this study was to assess whether CHD prolongs the time to achieve full enteral feeding in neonates underwent abdominal surgery.

**Methods:**

This was a dual-center retrospective study. Neonates who underwent abdominal surgery were subdivided into two groups: those with CHD and those without. The primary outcome was the time to achieve full enteral feeding after abdominal surgery. Propensity score matching (ratio of 1:2) was performed to balance covariates. *Cox* regression analysis was used to evaluate the association. Subgroup analysis further stratified CHD patients based on hemodynamic significance as determined by echocardiographic criteria.

**Results:**

A total of 341 newborns were enrolled: 136 with CHD and 205 without. For infants with vs. without CHD, the median gestational age was 35.6 (32.4, 37.8) weeks vs. 36.6 (32.1, 39.0) weeks, birth weight was 2270.0 (1547.5, 3007.5) vs. 2630.0 (1700.0, 3300.0) g, corrected gestational age on surgery day was 37.2 (34.2, 39.4) weeks vs. 39.0 (35.1, 41.4) weeks, respectively (all *P* < 0.05). There was no significant difference between 2 groups in preoperative feeding nor in operation duration (both *P* > 0.05). After propensity score matching, 64 neonates with CHD and 128 without CHD were compared. The postoperative time to achieve full feeding was 21.6 days (*95% CI*: 15.1 ~ 28.1) for CHD and 17.4 (*95% CI*: 14.2 ~ 20.7) days for non-CHD (*P* = 0.014). Multivariable analysis found that CHD was an independent risk factor (*HR* = 2.046, *95% CI*: 1.189 ~ 3.519, *P* = 0.010). Subgroup analysis revealed that only hemodynamically significant CHD (*HR* = 2.712, *95% CI*: 1.414 ~ 5.198, *P* = 0.003) contributed to this association, while non-significant CHD did not (*HR* = 1.112, *95% CI*: 0.523 ~ 2.370, *P* = 0.781).

**Conclusions:**

The presence of CHD significantly prolongs the time to achieve full enteral feeding postoperatively and increases the risk of feeding intolerance in neonates undergoing abdominal surgery, an effect primarily driven by hemodynamically significant CHD.

**Trial registration:**

This study was registered in the Chinese Clinical Trial Registry on November 8, 2025 (Registry website: http://www.chictr.org.cn) (Registration No. ChiCTR2500112685).

**Supplementary Information:**

The online version contains supplementary material available at 10.1186/s13052-026-02291-w.

## Background

Neonates in the intensive care unit (NICU) may encounter surgical issues, such as necrotizing enterocolitis [1] or gastrointestinal malformations [2]. These infants have a high risk of negative outcomes after surgery [3, 4], especially those with congenital heart disease (CHD) [5]. Surgical neonates with CHD have significantly higher mortality (22.2% vs. 1.3% in non-CHD neonates; *OR* = 17.1), mainly due to severe CHD-related complications, such as hemodynamic collapse and respiratory failure, rather than direct surgical risks like anastomotic leak or infection [6]. Postoperative morbidity rates are also significantly higher in children with CHD undergoing non-cardiac surgery. Complications include cardiovascular issues (e.g., arrhythmias, hypotension, pulmonary hypertensive crisis), respiratory problems (e.g., respiratory failure, extubation failure), acute kidney injury, and thromboembolic events, such as deep vein thrombosis and pulmonary embolism [7]. These outcomes are related to altered hemodynamics from CHD [8]. In healthy newborns, splanchnic blood flow is less than 5% of cardiac output when fasting, but rises to 30% after feeding to support digestion and absorption [9]. In neonates with CHD, intestinal perfusion does not adjust well to the increased demand after feeding. This situation worsens in neonates after abdominal surgery, which further impairs function and causes feeding intolerance [10]. Although CHD’s negative effect on surgical outcomes is known, its impact on postoperative nutritional recovery has not been well measured [11]. We hypothesize that the presence of CHD delays the establishment of full enteral feeding in neonates after abdominal surgery, resulting in prolonged hospital stays and increased disease burden. Our study aims to determine if CHD prolongs the time to full enteral feeding in neonates undergoing abdominal surgery.

## Methods

### Study design and setting

This was a dual-center, retrospective, observational study. The primary aim of this study was to investigate whether CHD prolongs the time to full enteral feeding in neonates after abdominal surgery. This study was approved by the Institutional Ethics Committee of Beijing Children’s Hospital, Capital Medical University (Approval No. [2025]-E-160-R) and registered in the Chinese Clinical Trial Registry (Registry website: http://www.chictr.org.cn) (Registration No. ChiCTR2500112685).

### Participants

Patients were hospitalized in neonatal intensive care units of 2 tertiary children’s hospitals: Beijing Children’s Hospital (70 beds) and Zhengzhou Children’s Hospital (50 beds). The two centers had comparable bed capacities, physician-to-nurse ratios, nurse-to-patient ratios, patient populations, and disease spectrums. Therefore, data from both centers were pooled for subsequent analyses. Inclusion criteria included: (1) Age on admission ≤ 28 days (for term infants) or corrected gestational age ≤ 44 weeks (for preterm infants); and (2) Underwent abdominal surgery under general anesthesia during the hospitalization. Exclusion criteria included: (1) Missing critical clinical data; or (2) Diagnosed with major congenital anomalies other than CHD or the primary abdominal condition (e.g., severe neurological malformations, chromosomal abnormalities) or inherited metabolic diseases. Patients were then divided into two groups: those with CHD and those without CHD.

### Enteral feeding protocol

Both centers followed a largely standardized enteral feeding protocol for neonates undergoing abdominal surgery. Feeding was initiated as soon as clinically feasible, typically within 24 ~ 48 h post-surgery. The initial feeding volume ranged from 10 to 20 mL/kg/d, depending on the neonate’s birth weight and preoperative feeding status. Feeds were gradually advanced by 10 ~ 20 mL/kg/d every 1 ~ 3 days, based on tolerance. Feeding tolerance was assessed using routine clinical parameters, including abdominal distension, gastric residuals, and vomiting. Feeding advancement was withheld or delayed in the presence of signs suggestive of intolerance or clinical instability. Full enteral feeding was defined as outlined in the Definitions section. While minor variations in day-to-day clinical decision-making may have occurred, there were no known systematic institutional differences in feeding strategies between the two centers.

### Definitions

Abdominal surgery [12] refers to surgical procedures that involve accessing the peritoneal cavity to diagnose, treat, or reconstruct intra-abdominal organs and structures via open or minimally invasive approaches, following the principles of safe access, sufficient exposure, tissue protection, and reliable abdominal wall closure. CHD refers to the structural or functional abnormality of the heart or great vessels present at birth [13]. Hemodynamically significant CHD refers to a structural cardiac abnormality detected by transthoracic echocardiography, including two-dimensional imaging, color Doppler, and spectral Doppler, that results in clinically or echocardiographically significant hemodynamic consequences. These include one or more of the following: (1) Cardiac chamber dilation or hypertrophy (e.g., right atrial or right ventricular enlargement suggesting a significant interatrial shunt); (2) Increased pressure gradient across a stenotic valve or vessel, while accounting for confounding factors such as heart rate and cardiac output; (3) Significant valvular regurgitation associated with systolic flow reversal in the systemic or pulmonary veins, or secondary chamber dilation; (4) A non-restrictive shunt or evidence of significant shunting leading to altered pulmonary-to-systemic blood flow (Qp/Qs); (5) Abnormal flow patterns in the aorta or pulmonary artery, such as holodiastolic flow reversal in the descending or abdominal aorta; (6) Estimated pulmonary artery systolic pressure ≥ 2/3 of systemic systolic pressure. Structural abnormalities that do not meet these criteria (e.g., small restrictive shunts, mild valvular stenosis without significant gradients, or trivial regurgitation without chamber dilation or flow abnormalities) are considered hemodynamically insignificant [14]. The critical illness status at admission was assessed using the NCIS [15]. Neonates were categorized as non-critical (NCIS > 90), critical (NCIS 70 ~ 90), or extremely critical (NCIS < 70). Full enteral feeding refers to a neonate’s daily enteral intake of at least 120 mL/kg/d [16]. Time to full enteral feeding after surgery was defined as the duration from the abdominal operation to the day when full enteral feeding was achieved. Weight gain velocity (g/kg/d) was calculated by dividing the total weight gain (in grams) by the number of days within a specific period, then dividing by the starting weight (kg) for that period.

### Data collection

Demographic data, Neonatal Critical Illness Score (NCIS) [15], discharge diagnoses, respiratory support, abdominal operation-related information, postoperative nutritional management and length of stay were collected from the hospital electronic medical record systems. The case report form and EpiData database were used.

### Statistical analysis

*SPSS* 25.0 was used to compare baseline characteristics of patients using univariate analyses: independent samples *t*-tests for normally distributed continuous variables, *Mann-Whitney U* tests for non-normally distributed ones, and *Chi-squared* or *Fisher’s exact* tests for categorical variables.

*Python* (version 3.13.3) with the pandas and scikit-learn libraries was used for propensity score matching. Steps included: (1) calculating propensity scores for each patient via logistic regression based on covariates with *P* < 0.1 in univariate analysis or clear clinical relevance; (2) performing 1:2 nearest-neighbor matching between CHD and non-CHD groups with a caliper width of 0.02; and (3) exporting the matched dataset for analysis. After matching, the *Kaplan-Meier curve* compared time to full enteral feeding between groups, with significance assessed by the *log-rank* test.

In the matched cohort, a multivariable *Cox* proportional hazards regression model was used to assess the independent association between CHD and time to full enteral feeding. The model included all covariates with *P* < 0.1 from the pre-matching univariate analysis to adjust for potential residual confounding. To determine whether CHD’s effect was attributable to hemodynamic severity, the CHD group was divided into hemodynamically significant and non-significant groups based on preoperative echocardiographic findings. The same *Cox* model, adjusted for the same covariates, was applied to assess the association between hemodynamic significance and time to full feeding. Results are reported as *Hazard Ratios* (*HR*) with 95% *Confidence Intervals* (*CI*).

## Results

### Demographics and clinical features before propensity score matching

Before propensity score matching, 341 newborns underwent abdominal surgery, of whom 136 had CHD and 205 did not. Demographics and clinical features before propensity score matching are listed in Table 1. For newborns with CHD who underwent abdominal surgery, gestational age, birth weight, corrected gestational age and weight on the day of surgery were significantly lower than those without CHD (all *P* < 0.05). There was no significant difference in NCIS scoring between the two groups (*P* = 0.942). The indications for surgical intervention are listed in Table 2, with no significant difference between the two groups (all *P >* 0.05). The top three indications for surgical intervention were intestinal obstruction due to congenital intestinal malformations, NEC and gastrointestinal perforation (excluding perforation of NEC) in both groups.

**Table 1:** Demographics and clinical features before propensity score matching

	With CHD (*n* = 136)	Without CHD (*n* = 205)	χ^2^	*P*
Male, *n* (%)	89 (65.4)	120 (58.5)	1.643	0.200
Singleton, n (%)	113 (83.1)	155 (75.6)	2.718	0.099
Preterm, n (%)	84 (61.8)	112 (54.6)	1.701	0.192
Gestational age, weeks^b^	35.6 (32.4, 37.8)	36.6 (32.1, 39.0)	-2.029	0.042
Birth weight, grams^b^	2270.0 (1547.5, 3007.5)	2630.0 (1700.0, 3300.0)	-2.329	0.020
Apgar score at 5 min ≤ 7, n (%)	4 (2.9)	3 (1.5)	0.888	0.346
Fetal distress, n (%)	21 (15.4)	23 (11.2)	1.297	0.255
Small for gestational age, n (%)	22 (16.2)	22 (10.7)	2.157	0.142
Extrauterine growth restriction at admission, n (%)	45 (33.1)	65 (31.7)	0.071	0.789
Critical illness severity at admission, by NCIS				
Non-critical, n (%)	68 (50.0)	104 (50.7)	0.120	0.942
Critical, n (%)	61 (44.9)	89 (43.4)		
Extremely critical, n (%)	7 (5.1)	12 (5.9)		
Preoperative intubation, n (%)	48 (35.3)	68 (33.2)	0.164	0.685
Preoperative achievement of full enteral feeding, n (%)	8 (5.9)	17 (8.3)	0.699	0.403
Corrected age at surgery, weeks^b^	37.2 (34.2, 39.4)	39.0 (35.1, 41.4)	-3.352	0.001
Weight at surgery, grams^a^	2451.8 ± 825.1	2760.7 ± 880.4	-3.252	0.001
Operative time, hours^b^	1.35 (0.92, 1.80)	1.48 (1.02, 1.95)	-1.515	0.130

^a^ Normally distributed continuous data are presented as Mean ± SD

^b^ Non-normally distributed continuous data are presented as Median (Q1, Q3)

Abbreviations: CHD, Congenital heart disease; NCIS, Neonatal Critical Illness Score

**Table 2:** Surgical indications before propensity score matching, n (%)

	With CHD (*n* = 136)	Without CHD (*n* = 205)	χ^2^	*P*
Intestinal obstruction				
Total intestinal obstruction	67 (49.3)	87 (42.4)	1.538	0.215
Caused by congenital intestinal malformations	60 (44.1)	75 (36.6)	1.940	0.164
Caused by intestinal malrotation	7 (5.1)	12 (5.9)	0.078	0.781
NEC				
Total NEC	37 (27.2)	60 (29.3)	0.171	0.679
With perforation	22 (16.2)	24 (11.7)	1.399	0.237
Without perforation	15 (11.0)	36 (17.6)	2.742	0.098
Non-NEC Gastrointestinal Perforation	19 (14.0)	34 (16.6)	0.426	0.514
Other indications	13 (9.6)	24 (11.7)	0.390	0.532

Data are presented as n (column %) within each group. Subcategories under “Total intestinal obstruction” and “Total NEC” are nested within their respective total category

Abbreviations: CHD, Congenital heart disease; NEC, Necrotizing enterocolitis

### Demographics and clinical features after propensity score matching

Covariates with *P* values < 0.1 in Table 1, along with the surgical site and preoperative infection status [17], were selected as confounding factors for propensity score matching. Next, 64 neonates with CHD were matched to 128 without CHD. After matching, all covariates had *P* values > 0.05 and standardized mean differences (SMD) < 0.25, indicating that the two groups were well balanced (Table 3).

**Table 3:** Demographics and clinical features after propensity score matching

	With CHD (*n* = 64)	Without CHD (*n* = 128)	χ^2^	*P*	SMD
Singleton, *n* (%)	51 (79.7)	99 (77.3)	0.137	0.711	0.057
Gestational age, weeks^b^	35.6 (32.4, 37.8)	35.0 (31.3, 38.7)	-0.008	0.993	0.065
Birth weight, grams^b^	2395.0 (1642.5, 3152.5)	2265.0 (1573.8, 3150.0)	-0.176	0.860	0.103
Corrected age at surgery, weeks^a^	37.19 ± 3.92	37.71 ± 4.03	-0.859	0.391	0.131
Weight at surgery, grams^a^	2591.25 ± 910.17	2520.00 ± 749.11	0.069	0.945	0.086
No infection before abdominal operation, n (%)	15 (23.4)	29 (22.6)	0.015	0.903	0.018
Confirmed infection before abdominal operation, n (%)					
Sepsis complicated with purulent meningitis	27 (42.1)	53 (41.4)	0.011	0.918	0.016
Sepsis only	8 (12.5)	11 (8.6)	0.730	0.393	0.127
Pneumonia	6 (9.4)	15 (11.7)	0.241	0.624	-0.076
Sepsis complicated with peritonitis	3 (4.7)	6 (4.7)	0.000	1.000	0.000
Other infections	2 (3.1)	9 (7.0)	0.591	0.442	-0.178
NEC	3 (4.8)	5 (3.9)	0.000	1.000	0.038
Abdominal operation site, n (%)					
Small intestine	53 (82.8)	110 (85.9)	0.325	0.569	-0.086
Colon-rectum	6 (9.38)	12 (9.38)	0.000	1.000	0.000
Appendix	2 (3.13)	2 (1.56)	0.511	0.602	0.104
Hepatobiliary system	1 (1.56)	2 (1.56)	0.000	1.000	0.000
Abdominal wall	2 (3.13)	2 (1.56)	0.511	0.602	0.104

^a^ Normally distributed continuous data are presented as Mean ± SD

^b^ Non-normally distributed continuous data are presented as Median (Q1, Q3)

Balance after matching was assessed using both P value and SMD. *P* > 0.05 indicates no significant difference, and SMD < 0.25 indicates acceptable balance

Abbreviations: CHD, Congenital heart disease; NEC, Necrotizing enterocolitis

### Kaplan-meier curves after propensity score matching

**Fig. 1:**
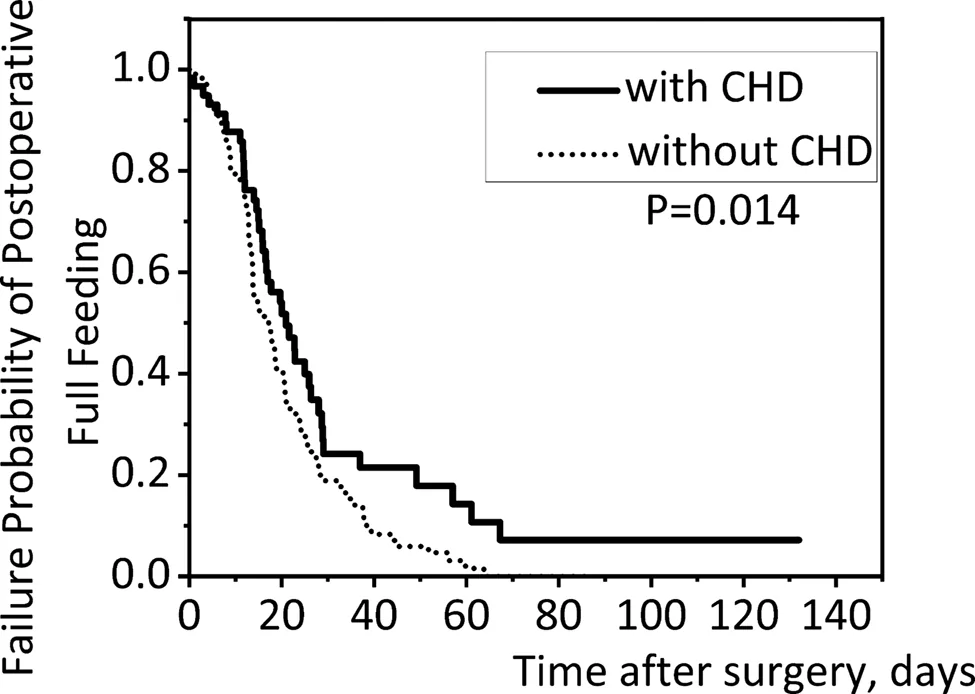
*Kaplan-Meier Curve* for the Failure Probability of Postoperative Full Feeding. The X and Y axis represent time after abdominal surgery and the probability of not achieving postoperative full enteral feeding. A probability of 1.0 indicates that no neonates had achieved the endpoint at time zero. Both curves showed a declining trend in neonates who underwent abdominal operations, with the trend declining less rapidly in infants with CHD (thick solid line) than in those without CHD (thin dotted line). The difference between the curves was analyzed using the *log-rank test*, which showed a statistically significant difference (*P* = 0.014). Abbreviation: CHD, congenital heart disease

After propensity score matching, the *Kaplan-Meier* curve for postoperative full feeding is shown in Fig. 1. Infants with CHD had a median time of 21.6 days (*95% CI*: 15.1 ~ 28.1) to resume full enteral feeding, while those without CHD had a median time of 17.4 days (*95% CI*: 14.2 ~ 20.7). The *Log-rank* test yielded a *χ²* of 6.019 (*df* = 1, *P* = 0.014).

### Cox regression analysis after propensity score matching

After propensity score matching, univariable *Cox* regression analysis was performed to identify covariates associated with the time to full enteral feeding (Table 4). Multivariable analysis was then conducted for covariates with *P* < 0.1 in Table 4. In the model treating CHD as a binary variable, CHD remained an independent risk factor for prolonged postoperative time to full feeding (*HR* = 2.046, *P* = 0.010, *95% CI*: 1.189 ~ 3.519), along with vasoactive drug use (*P* = 0.005) and electrolyte imbalance (*P* = 0.008) (Table 5). Notably, when CHD was further stratified by hemodynamic significance based on echocardiographic criteria, 39 neonates were classified as having hemodynamically significant CHD and 25 as hemodynamically non-significant CHD. In this subgroup analysis, only hemodynamically significant CHD was independently associated with delayed feeding (*HR* = 2.712, *95% CI*: 1.414 ~ 5.198, *P* = 0.003), indicating a stronger impact on postoperative feeding time. In contrast, hemodynamically non-significant CHD showed no such association (*HR* = 1.112, *95% CI*: 0.523 ~ 2.370, *P* = 0.781) (Table 6). In addition, perioperative vasoactive agent use was identified as an independent predictor of prolonged time to full enteral feeding (*HR* = 2.242, *95% CI*: 1.298 ~ 3.874, *P* = 0.004).

**Table 4:** Univariable *Cox* regression analysis after propensity score matching

Variable	b	Wald	*P*	HR	95% CI
Preterm	1.013	33.496	<0.001	2.783	1.968 ~ 3.935
Electrolyte imbalance	0.969	28.780	<0.001	2.636	1.850 ~ 3.755
Steroid therapy	0.907	6.106	0.013	2.476	1.206 ~ 5.083
Hypotension	0.808	19.740	<0.001	2.244	1.571 ~ 3.205
Blood product transfusion	0.740	18.828	<0.001	2.096	1.489 ~ 2.949
Birth weight, kg	0.637	44.879	<0.001	1.891	1.569 ~ 2.278
Vasoactive drug use	0.629	11.996	0.001	1.877	1.314 ~ 2.680
With CHD	0.459	5.921	0.015	1.583	1.093 ~ 2.292
Weight at surgery, kg	0.373	19.376	<0.001	1.453	1.230 ~ 1.716
Metabolic acidosis	0.355	3.325	0.068	1.426	0.974 ~ 2.087
Singleton	0.272	1.765	0.184	1.313	0.879 ~ 1.962
Corrected age at surgery	0.114	27.517	<0.001	1.121	1.074 ~ 1.170
Peak lactate	0.089	2.368	0.124	1.093	0.976 ~ 1.222
Fetal distress	0.088	0.146	0.702	1.092	0.695 ~ 1.716
White blood cell count	0.026	3.495	0.062	1.026	0.999 ~ 1.054
Postoperative peak FiO_2_	0.010	0.977	0.323	1.010	0.990 ~ 1.030
Peripheral hemoglobin level at surgery	0.009	13.241	<0.001	1.009	1.004 ~ 1.013
Postoperative IMV duration	0.005	11.272	<0.001	1.005	1.002 ~ 1.009
Maternal diabetes	-0.113	0.282	0.596	0.893	0.588 ~ 1.356
Male	-0.121	1.937	0.164	0.886	0.747 ~ 1.051
Hypoglycemia	-0.462	1.976	0.160	0.630	0.330 ~ 1.200
Preoperative hypothermia	-0.476	1.471	0.225	0.621	0.288 ~ 1.341
Apgar score at 5 min < = 7	-1.076	2.238	0.135	0.341	0.083 ~ 1.396

Abbreviations: CHD, Congenital heart disease; IMV, Invasive mechanical ventilation; FiO_2_, Fraction of inspired oxygen; PSM, Propensity Score-Matched Cohort

**Table 5:** Multivariable *Cox* regression analysis after propensity score matching

Variable	b	Wald	*P*	HR	95% CI
With CHD	0.716	6.688	0.010	2.046	1.189 ~ 3.519
Vasoactive drug use	0.772	7.825	0.005	2.165	1.260 ~ 3.719
Electrolyte imbalance	0.652	6.970	0.008	1.919	1.183 ~ 3.112
Steroid therapy	0.462	0.801	0.371	1.587	0.577 ~ 4.366
Birth weight, kg	0.272	1.124	0.289	1.313	0.793 ~ 2.173
Blood product transfusion	0.165	0.355	0.551	1.179	0.686 ~ 2.026
Corrected age at surgery	0.098	3.497	0.061	1.103	0.995 ~ 1.222
Hypotension	0.059	0.044	0.834	1.061	0.610 ~ 1.847
Preterm	0.033	0.008	0.931	1.034	0.489 ~ 2.185
Metabolic acidosis	0.026	0.009	0.926	1.026	0.600 ~ 1.755
White blood cell count	0.023	0.968	0.325	1.024	0.977 ~ 1.073
Peripheral hemoglobin level at surgery	0.005	1.881	0.170	1.005	0.998 ~ 1.013
Postoperative IMV duration	0.000	0.001	0.981	1.000	0.997 ~ 1.003
Weight at surgery, kg	-0.128	0.226	0.570	0.879	0.564 ~ 1.370

Abbreviations: CHD, Congenital heart disease. IMV, Invasive mechanical ventilation; PSM, Propensity Score-Matched Cohort

**Table 6:** Multivariable *Cox* regression analysis of CHD severity grade for time to full enteral nutrition

Variable	b	Wald	*P*	HR	95% CI
Hemodynamically significant CHD	0.998	9.026	0.003	2.712	1.414 ~ 5.198
Vasoactive drug use	0.808	8.380	0.004	2.242	1.298 ~ 3.874
Electrolyte imbalance	0.714	8.394	0.004	2.042	1.260 ~ 3.311
Steroid therapy	0.494	0.902	0.342	1.639	0.591 ~ 4.540
Birth weight, kg	0.353	1.828	0.176	1.424	0.853 ~ 2.376
Blood product transfusion	0.210	0.580	0.446	1.233	0.719 ~ 2.115
Hemodynamically Non-significant CHD	0.107	0.077	0.781	1.112	0.523 ~ 2.370
Corrected age at surgery	0.099	3.700	0.054	1.104	0.998 ~ 1.222
Preterm	0.083	0.044	0.834	1.087	0.498 ~ 2.372
Hypotension	0.054	0.036	0.850	1.055	0.605 ~ 1.840
White blood cell count	0.025	1.109	0.292	1.025	0.979 ~ 1.075
Peripheral hemoglobin level at surgery	0.004	1.222	0.269	1.004	0.997 ~ 1.012
Postoperative IMV duration	0.000	0.015	0.902	1.000	0.977 ~ 1.003
Metabolic acidosis	-0.017	0.004	0.951	0.983	0.574 ~ 1.685
Weight at surgery, kg	-0.299	1.502	0.220	0.741	0.459 ~ 1.196

Abbreviations: CHD, Congenital heart disease. IMV, Invasive mechanical ventilation; PSM, Propensity Score-Matched Cohort

## Discussion

In this propensity score-matched study of 341 neonates, CHD was associated with prolonged postoperative time to full enteral feeding and an increased risk of feeding intolerance. This association was primarily driven by hemodynamically significant CHD, whereas non-significant CHD showed no independent association in the matched cohort. These findings support a more cautious and gradual enteral feeding approach in patients with CHD who underwent abdominal surgery. Our results are consistent with a recent review indicating that CHD, particularly severe subtypes, is associated with delayed achievement of full enteral feeding [11].

The difference in median time to full enteral feeding between the CHD and non-CHD groups (approximately 4 days) was statistically significant and clinically meaningful. It directly delays the transition from parenteral to enteral feeding, with important implications for the neonate’s recovery. Prolonged dependence on parenteral nutrition increases the risk of complications such as catheter-related bloodstream infections, sepsis, venous thrombosis, and parenteral nutrition-associated liver disease [18]. Furthermore, the delay in achieving full enteral feeding can extend mechanical ventilation duration, ICU stay, and overall hospitalization, potentially contributing to increased postoperative morbidity [11]. Even in the absence of major complications, short-term nutritional deficits, such as protein malnutrition, can have cumulative effects, including increased postoperative infection risk and delayed wound healing, further hindering recovery [19].

The prolonged time to full enteral feeding in neonates with congenital heart disease (CHD) after abdominal surgery results from both cardiac and surgical factors. These factors initiate pathological mechanisms that directly diminish intestinal tolerance to enteral feeding. As a consequence, clinicians often need to advance feeding more slowly, which ultimately extends the time needed to reach full enteral feeding.

In neonates with CHD, reduced cardiac output from structural defects like single ventricle, hypoplastic left heart syndrome, or tetralogy of Fallot redistributes blood. More blood goes to the heart and brain; less reaches the gut. In infants with a patent ductus arteriosus or intracardiac shunting, blood shifts from the systemic to the pulmonary circulation, decreasing intestinal perfusion. Severe shunting can cause retrograde flow from the mesenteric artery to the aorta, worsening gut perfusion [20–22]. Neonates with hypoplastic left heart syndrome may have mesenteric vascular issues, predisposing them to hypoperfusion [23]. CHD often activates the renin-angiotensin-aldosterone system (RAAS), constricting mesenteric vessels and worsening intestinal ischemia. It also triggers the NF-κB pathway, which promotes inflammation and impairs mucosal repair [24]. Intestinal hypoperfusion leads to ischemic mucosal injury, villous atrophy, and increased intestinal permeability, impeding nutrient absorption and slowing feeding progression. Chronic hypoxemia further damages intestinal epithelial cells and disrupts the gut barrier, making feeding harder [21].

Surgical and iatrogenic factors also contribute. Abdominal surgery results in direct intestinal injury, including mucosal damage, disrupted enteric nerves, and smooth muscle injury [25]. Some medications used for CHD management, such as indomethacin, can inhibit gastrointestinal motility, while certain vasoactive agents used to maintain blood pressure, like dopamine and epinephrine, may reduce mesenteric blood flow and impair intestinal motility [26]. Notably, our multivariable analysis further confirmed the independent impact of vasoactive agent use: perioperative vasoactive support was identified as a strong independent predictor of delayed full enteral feeding (*HR* = 2.242, *95% CI*: 1.298 ~ 3.874, *P* = 0.004). This observation supports a potential role of intestinal hypoperfusion in feeding delay and suggests a more cautious approach to feeding advancement in neonates requiring vasoactive agents.

While these pathophysiological mechanisms provide a plausible explanation for delayed enteral feeding in neonates with CHD, it is important to consider whether the observed association could instead be attributed to baseline differences between groups. Although differences in birth weight and gestational age were present prior to matching, these were largely balanced after propensity score matching. This suggests that the association is unlikely to be fully explained by these factors.

Our findings are broadly consistent with prior reports by Faraoni et al. and Chu et al., which demonstrated an increased risk of mortality and postoperative complications in children with CHD undergoing non-cardiac surgery [5, 27, 28]. Rather than focusing on these downstream outcomes, our study identifies delayed enteral feeding as an earlier and clinically relevant manifestation of postoperative recovery in this population [29–32].

By specifically examining neonates and applying propensity score matching and multivariable adjustment, we provide a more granular assessment of recovery that is less likely to be confounded by baseline characteristics. Taken together, these findings suggest that the observed delay in achieving full enteral feeding is more likely related to CHD-associated hemodynamic vulnerability, rather than baseline differences alone, and highlight feeding recovery as a meaningful intermediate outcome in this high-risk group.

This study has several limitations. First, its retrospective design introduces information bias due to reliance on medical records. Although both centers followed a largely standardized feeding protocol, retrospective data collection may not have captured transient deviations, such as temporary interruptions due to feeding intolerance. However, given the overall consistency in feeding practices and close inter-center collaboration, the impact of such variability is likely to be limited. Second, although we employed propensity score matching (PSM) to control for known confounders, residual bias from unmeasured variables, such as subtle variations in clinical decision-making and family socioeconomic status, cannot be ruled out [33]. Third, despite stratifying CHD by hemodynamic significance, residual heterogeneity within each group may persist, potentially limiting the precision of subgroup interpretation [7, 34, 35]. Fourth, the matched cohort was relatively small, particularly in the CHD group. This limited statistical power may have affected the effect estimates for certain covariates (e.g., electrolyte imbalance) in the multivariable analysis and restricted meaningful subgroup analyses within the hemodynamically significant and non-significant CHD subgroups. Finally, as both participating institutions are Tertiary Grade A children’s hospitals with advanced multidisciplinary expertise, the generalizability of our findings to other clinical settings may be limited. Further validation through robust, prospective, multicenter studies with larger samples is necessary.

## Conclusion

This study demonstrates that congenital heart disease (CHD) is associated with a prolonged time to full enteral feeding after abdominal surgery. Subgroup analysis using multivariable *Cox* regression further suggests that this association is primarily driven by hemodynamically significant CHD, whereas non-significant CHD shows no independent effect. Neonates with hemodynamically significant CHD may benefit from a more cautious and individualized approach to enteral feeding advancement. Future prospective multicenter studies with larger sample sizes are warranted to validate these findings and to inform the development of nutritional strategies for this population.

## Supplementary Information

Below is the link to the electronic supplementary material.

Supplementary Material 1

## Data Availability

To protect participant privacy, the individual data supporting this study cannot be made publicly available. De-identified data may be shared with qualified researchers upon reasonable request to the corresponding author, Mingyan Hei, subject to a formal data use agreement.

## Abbreviations

NICU: Neonatal intensive care unit
CHD: Congenital heart disease
NCIS: Neonatal Critical Illness Score
PSM: Propensity score matching
FiO_2_: Fraction of inspired oxygen
IMV: Invasive mechanical ventilation
NEC: Necrotizing enterocolitis
